# The prion-like transmission of tau oligomers *via* exosomes

**DOI:** 10.3389/fnagi.2022.974414

**Published:** 2022-08-18

**Authors:** Noel A. Jackson, Marcos J. Guerrero-Muñoz, Diana L. Castillo-Carranza

**Affiliations:** ^1^School of Public Health, Harvard University, Boston, MA, United States; ^2^School of Medicine, University of Monterrey, San Pedro Garza García, Mexico

**Keywords:** tau oligomers, exosomes, vesicles, misfolding, spreading, prion

## Abstract

The conversion and transmission of misfolded proteins established the basis for the prion concept. Neurodegenerative diseases are considered “prion-like” disorders that lack infectivity. Among them, tauopathies are characterized by the conversion of native tau protein into an abnormally folded aggregate. During the progression of the disease, misfolded tau polymerizes into oligomers and intracellular neurofibrillary tangles (NFTs). While the toxicity of NFTs is an ongoing debate, the contribution of tau oligomers to early onset neurodegenerative pathogenesis is accepted. Tau oligomers are readily transferred from neuron to neuron propagating through the brain inducing neurodegeneration. Recently, transmission of tau oligomers *via* exosomes is now proposed. There is still too much to uncover about tau misfolding and propagation. Here we summarize novel findings of tau oligomers transmission and propagation *via* exosomes.

## Introduction

Tauopathies, such as Alzheimer’s disease (AD) and Parkinson’s disease dementia (PDD), are neurodegenerative disorders involving the spread of toxic forms of tau protein, resulting in characteristic aggregates and cytoplasmic inclusions that impair normal neuronal functioning (Irwin et al., 2013; Shi et al., 2016; Orr et al., 2017; Polanco et al., 2018b). In normal states, tau’s primary function is to stabilize microtubules and is mainly found in neuronal axons (Binder et al., 1985; Shi et al., 2016) and the nuclear compartment (Bukar Maina et al., 2016). Through dynamic interactions with tubulin, tau promotes polymerization and stability of axons, neurite polarity, axonal sprouting and neuroplasticity (Samsonov et al., 2004). These functions are impaired in tauopathies (Lee et al., 2001; Binder et al., 2005; Goedert and Jakes, 2005).

In pathological states, native, unfolded tau isoforms are hyperphosphorylated and abnormally aggregate into a variety of soluble and insoluble conformations, such oligomers (multimers of tau) and neurofibrillary tangles (NFTs), respectively. Misfolding and aggregation of tau into oligomers is a major event in the pathogenesis of tauopathies. Studies have shown that oligomeric forms of tau are toxic to neurons (Lasagna-Reeves et al., 2012b; Usenovic et al., 2015; Ozcelik et al., 2016) and capable of spreading from neuron to neuron (Liu et al., 2012; Dujardin et al., 2014b; Jiang et al., 2020). While hyperphosphorylated NFTs are referred to as the pathological hallmark that define neurodegenerative tauopathies, the toxic properties of insoluble NFTs is questionable (Gomez-Isla et al., 1997; Terry, 2000; Wittmann et al., 2001; Spires-Jones et al., 2011). For instance, the aggregation and subsequent uptake of pre-fibrillar tau is associated with neuronal dysfunction before NFT formation. Contrary to NFTs, tau oligomers have been found in the extracellular space (Hyman, 2014; Puangmalai et al., 2020), cerebrospinal fluid (CSF) (Sengupta et al., 2017), and serum of AD patients (Kolarova et al., 2017). These extracellular tau oligomers are capable of seeding tau aggregation and propagation to neighboring cells affecting various neuronal functions including axonal transport, synaptic transmission, mitochondrial and endoplasmic reticulum function (Gerakis and Hetz, 2018; Polanco et al., 2018a), and chromatin opening within the nucleus (Frost et al., 2014). Although the role of tau oligomers into the mechanism of the disease and cell-to-cell transmission is not understood, recent studies have suggested that tau transmission may occur *via* exosomes (Polanco et al., 2016, 2018b,^2021^; Miyoshi et al., 2021). These are vesicles that seemingly provide the condition for protein aggregation and a vehicle to transfer tau oligomers between cells. Delivered tau oligomers may act as seeds, inducing the misfolding of native tau into a toxic conformation in recipient cells. Tau’s conversion and further propagation resembles the mechanism of prions. The basis of the prion-like concept are the seeded aggregation and propagation of misfolded proteins through the brain. Similar to prions, tau oligomers self-propagate, and spread from neuron-to-neuron and through the CNS (Bellingham et al., 2012a). Like prions, tau is transferred between cells *via* exosomes (Fevrier et al., 2004; Wang et al., 2017). Nevertheless, the mechanisms governing the transfer of tau have yet to be determined. In this review article we summarize recent findings of the prion-like transmission of tau oligomers *via* exosomes.

## Protein tau

Tau, an intrinsically disordered protein that lacks a tertiary structure (Uversky, 2018), can adopt a wide variety conformations. The gene encoding tau protein produce six isoforms containing three repeats (3R) or four repeats (4R), by alternative splicing of exons 2, 3, and 10 (Goedert et al., 1989). 1N4R tau is enriched in the nuclear fraction of brain lysates (Liu and Gotz, 2013). During disease, the imbalance of tau isoforms affects microtubule binding affinity. Misfolded tau isoforms deposit into distinct aggregates such oligomer, paired helical and straight filaments and NFTs (Fitzpatrick et al., 2017). Although, the differential expression of tau isoforms across tauopathies is not clear, cross-seeding between tau isoforms seems to be due to an asymmetric seeding barrier similar to prion transmission among species (Kumar and Udgaonkar, 2018; Weismiller et al., 2018).

## Tau oligomers

**FIGURE 1:**
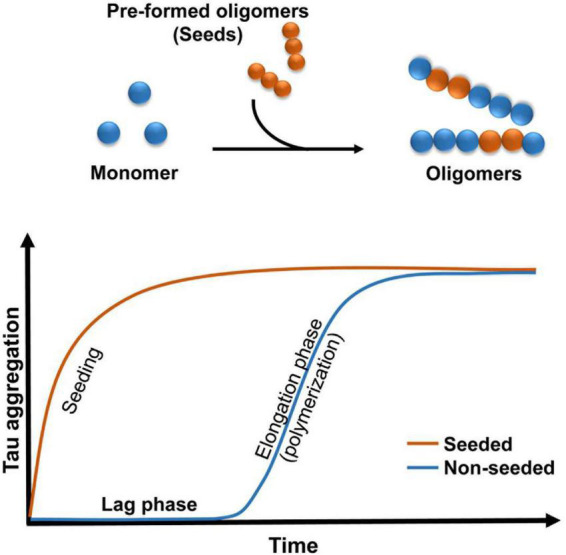
Schematic illustrating the kinetics of oligomers formation. Addition of pre-formed oligomers reduced the lag phase.

During the pathogenesis of tauopathies, functional tau loses affinity for microtubules and self-aggregates into a mixture of soluble and insoluble structures that vary in size. Tau oligomers are low molecular weight polymers comprised of a small number of repeating tau protein units. These forms serve as an intermediate conformation between tau monomers and fibrils (Figure 1). Although a large body of evidence suggest that tau oligomers are the toxic species (Lasagna-Reeves et al., 2011, 2012a; Castillo-Carranza et al., 2014a,b, 2018; Gerson et al., 2016), other studies argue that fibrils display toxic properties (Guo and Lee, 2011; Iba et al., 2013). NFTs were initially considered the toxic species responsible for neuronal loss. Recent studies have provided clear evidence that toxicity of tau fibrils depends upon its breakdown into small soluble oligomers and short fibrils. For instance, sonication of tau fibrils enhanced cell toxicity *in vitro* (Ghag et al., 2018). Moreover, *in vivo* and *in vitro* studies showed that oligomeric tau, not fibrillar tau, is physiologically active given the capacity to induce translational stress response (Jiang et al., 2020). A likely explanation is that these newly formed oligomers are easily internalized by neighboring cells to disrupt cellular homeostasis.

Tau oligomer accumulation has been shown in several tauopathies. In AD and progressive supra nuclear palsy (PSP), the presence of tau oligomers is associated with the onset of clinical symptoms of the disease (Lasagna-Reeves et al., 2012b). Coincidently, increased levels of tau oligomers in CSF and serum from AD patients predicts worst clinical outcome (Wallin et al., 2010; Sengupta et al., 2017). The molecular changes in AD are reflected in CSF. Altered CSF composition may lead to vulnerability of neurons. In dominant AD, tau levels increased before the onset of the disease (Bateman et al., 2012), suggesting a deregulation in tau expression prior to the aggregated pathological conditions.

Consistent with the effects driven by tau oligomers in the brain, recombinant counterparts exert toxicity *in vivo*. In mice, administration of brain-derived (Lasagna-Reeves et al., 2012a; Castillo-Carranza et al., 2015) as well as recombinant tau oligomers induced cognitive, synaptic, and mitochondrial abnormalities (Lasagna-Reeves et al., 2011; Patterson et al., 2011; Sydow et al., 2011). Conversely, the removal of tau oligomers by immunotherapy has proven beneficial in mice models of tauopathies, by reversing memory deficits associated to tau pathology (Castillo-Carranza et al., 2014a,b, 2015).

## The prion-like transmission of tau oligomers

The intercellular transmission and propagation of misfolded forms of tau share many features with prions. For instance, misfolded prions and tau, can act as seeds that induce the conversion of poorly-structured monomeric protein into a β-sheet rich structures that further propagate in the brain (Brundin et al., 2010; Guo et al., 2016). Prion diseases are a distinct group characterized by the transmission of misfolded prion agents that interact with functional proteins to induce misfolding. Neurodegenerative prion diseases that afflict humans include Creutzfeldt-Jakob disease (CJD) and Gertsmann-Straussler-Scheinker syndrome (GSS) (Aguzzi and Heikenwalder, 2006). Prions, the transmissible agent in these diseases, are proteins with high resistance to chemical and physical methods used to denature or degrade proteins (Cobb and Surewicz, 2009). The prions infect by interacting with functional proteins of a similar origin to induce their conversion. Contrary to neurodegenerative diseases, prion diseases are spread in an infectious manner between individuals.

Experiments conducted with brain homogenates from distinct tauopathies reproduce certain pathological features of the diseases in mice, which is consistent with the prion strain behavior (Clavaguera et al., 2009; Lasagna-Reeves et al., 2012a; Clavaguera et al., 2013; Ahmed et al., 2014; Sanders et al., 2014; Boluda et al., 2015). Evidence has shown that decreasing exposure to tau seeds results in a reduction in tau pathology *in vivo* (Castillo-Carranza et al., 2014a), suggesting that a pathological concentration threshold is required for the spread of tau aggregates (Baker et al., 2016). Tau may be released into extracellular space during cell death (Dujardin et al., 2014a), suggesting that tau may play a major role in the degenerative process (de Calignon et al., 2012; Liu et al., 2012). There are many current hypotheses on the process by which tau is released and spread. Extracellular tau plays a major role in the pathobiological aspects of these tauopathies. Evidence has shown that tau can potentiate toxicity when administered extracellularly (Gomez-Ramos et al., 2009). Additionally, tau oligomers may act as prions by inducing the misfolding of functional tau into oligomeric conformations (Soto and Estrada, 2008; Clavaguera et al., 2009; Novak et al., 2011; Wu et al., 2013). This “prion-like” behavior involves the transfer of tau oligomer seeds between cells and the further conversion of normal tau to generate new seeds (Figure 1). It is unclear how this process develops *in vivo*, but it is possible that misfolded proteins follow the “seeding-nucleation” model. During the initial slow lag phase only a limited number of misfolded proteins (seeds) are produced, and this is followed by a fast growing elongation phase (Kayed et al., 2003). Studies conducted in mice have shown that small amounts of intracerebrally infused recombinant and brain-derived oligomers propagated tau oligomer formation, behavioral deficits and neurodegeneration (Lasagna-Reeves et al., 2011, 2012a; Castillo-Carranza et al., 2014a,^2018^). It is possible that these small soluble intermediates might be easily internalized *via* bulk endocytosis in the recipient cell (Wu et al., 2013).

## Interaction of RNA binding proteins with tau oligomers

Yeast prion protein contained low complexity domains (LCDs), that assemble into a parallel cross-β structure. The LCDs were first identified in budding yeast (Fomicheva and Ross, 2021). LCDs promote cytoplasmic inclusions called stress granules. These are dynamic membraneless organelles containing RNA binding protein (RBPs) that form in response to stress in neurodegenerative diseases such amyotrophic lateral sclerosis (ALS). RBPs with LCDs are known to drive protein aggregation in neurodegenerative diseases. These proteins include protein fused in sarcoma (FUS) (Dormann et al., 2010), TAR DNA-binding protein-43 (TDP-43) (Cohen et al., 2011), T-cell intracellular antigen-1 (TIA1) (Vanderweyde et al., 2016), and Musashi (Sengupta et al., 2018) among others. In AD and other tauopathies, RBPs interact with tau leading to toxic aggregates. But how this interaction arises is unclear.

Many RBPs with PLDs contain nuclear localization signals (NLS) (King et al., 2012). Although none of the tau isoforms carry NLS, the protein tau executes a physiological nuclear function (Brady et al., 1995). Apparently, environmental conditions modulate neuronal transport of tau from cytoplasm to nucleus. The proline-rich domain of tau interacts with DNA (Sultan et al., 2011). In fact, during stress conditions tau protects DNA from reactive oxygen species (ROS) (Montalbano et al., 2021). Inside the nucleolus, tau is associated with the nucleolar organizer regions (Sjoberg et al., 2006), and given the functions of nucleolus, tau may influence RNA translation and ribosomal assembly.

In pathological conditions, phosphorylated tau inhibits trafficking of macromolecules between cytoplasm and nuclear compartment. In tau transgenic mice and AD brain, tau relocated from the nuclear membrane to the nuclear pore complexes (NPC), leading to cytoplasmic aggregates, comprised of phosphorylated tau and nuclear pore proteins (Eftekharzadeh et al., 2019). In *Drosophila* model of tauopathy and cells in culture, disease-associated forms of tau depletes nuclear Ca2+ inducing neuronal death (Mahoney et al., 2020). In neurons from brain cortex and hippocampus of early Braak stage AD, and in Huntington disease, tau deposit in a nuclear rodlet-shaped formation called tau nuclear rods (TNRs) (Fernandez-Nogales et al., 2014). TNRs consist of an invagination of the nuclear envelope filled with tau. In addition to large tau aggregates related to RBPs, oligomeric tau aggregates containing RNA-binding proteins impair chromatin remodeling and nuclear lamina formation in the nuclear compartment (Montalbano et al., 2020, 2021). In AD brain, tau oligomers interact with the RNA binding protein Musashi (MSI), whereas *in vitro* tau oligomers seeded MSI aggregation (Sengupta et al., 2018). MSI proteins enhanced tau nuclear translocation (Montalbano et al., 2019). Other RBPs that interact with tau oligomers include TIA1. Oligomeric tau inclusions in neuronal cytoplasm were colocalized with TIA1 and other stress granules proteins. Moreover, reducing TIA1 decreased neurodegeneration induced by propagated oligomeric tau (Jiang et al., 2019). Thus, given that RNA can also induce tau fibrillization (Kampers et al., 1996; Wang et al., 2006), concentrating tau protein, RNA and/or RBPs in a constrained space may result in tau’s aggregation inside cytoplasmic space or vesicles such exosomes.

In fact, there is evidence from AD and related tauopathies in which tau coincided with RBPs in early exosomes. For instance, in AD, tau pathology strongly correlates with cytoplasmic dense-cored granules called granulovacular bodies (GVB). These are intracellular compartment that contained different proteins in route to intracellular degradation. Granulovacuolar degeneration is a feature of pre-clinical AD in pre-tangle neurons that coincided with hippocampal phosphorylated tau accumulation (Nijholt et al., 2012). In transgenic mice, P301L and P301S tau pathology induced the formation of GVBs *in vivo* (Wiersma et al., 2019), suggesting that tau buildup triggers the formation of GVBs. Granulovacuolar degeneration bodies are neuron-selective lysosomal structures induced by intracellular tau pathology. GVBs contained RBPs such as TDP-43 and phosphorylated tau colocalized with exosomal marker Flotillin-1 (Yamoah et al., 2020). These finding suggest that vesicles such exosomes have an environment that promotes protein aggregation.

## Biogenesis of exosomes

A major question regarding the aggregation of tau and its contribution to neurodegeneration is how the protein is transported between cells. Exosomes have been studied for their role in neurodegenerative diseases as vehicles for the cell-to-cell transmission of the pathogenic proteins (Trams et al., 1981; Guo and Lee, 2014; Asai et al., 2015; Aryani and Denecke, 2016). These plasma membrane-derived nanovesicles have been proposed as a vehicle for seeding tau pathology throughout the CNS. Neurodegenerative diseases involve the aggregation and spread of toxic proteins through the brain. This suggests that the transfer of misfolded proteins contributes to the pathology of neurodegenerative disorder (Quek and Hill, 2017). One way this transfer could occur is through cell-derived exosomes. Due to their ability to diffuse across the blood brain barrier, exosomes are diagnostic in neurodegenerative conditions (Fiandaca et al., 2015; Lugli et al., 2015; Peskind et al., 2015). Many resident CNS cell types, including neurons, astrocytes, and glia, release exosomes into the extracellular environment (Bellingham et al., 2012b; Polanco et al., 2018b). These nanovesicles can deliver cargo, such as proteins, RBPs and RNAs, to neighboring cells.

**FIGURE 2:**
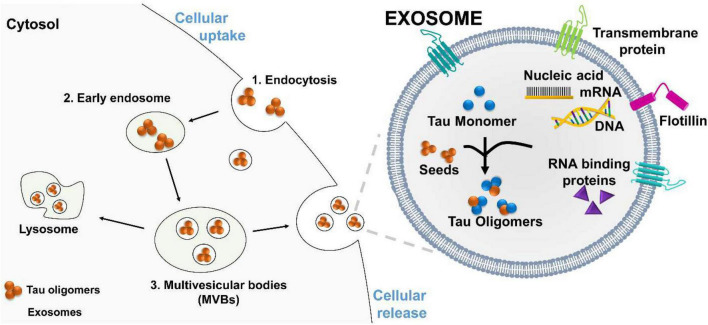
Proposed mechanism of tau oligomers spreading *via* exosomes. Exosomes are vesicles that carry RNA binding protein (RBP), nucleic acids, monomeric, and oligomeric tau. Exosomes facilitated oligomers formation and deliver their toxic cargo, to a recipient cell. (1) Tau oligomers are endocytosed by the recipient cell, (2) and transported to an early endosome, which develops into a large (3) multivesicular bodies (MVBs). Tau oligomers are release by fusion of MVBs with the plasma membrane.

Exosomes are plasma-membrane-derived nanovesicles ranging from 30 to 200 nm. Exosomes are released by most types of mammalian cells (Bellingham et al., 2012b; Polanco et al., 2018b). Initially, exosomes were thought to function solely as vehicles to remove biomolecular waste during the transition of reticulocytes to erythrocytes (Quek and Hill, 2017). These vesicles are produced from late endosomes by a process called endocytosis (Figure 2). The first step of endocytosis is the inward budding of the endosomal membrane, which develops accumulations of intraluminal nanovesicles (ILVs) (Mathivanan et al., 2010; Howitt and Hill, 2016; Boyiadzis and Whiteside, 2017). This late endosome develops into large multivesicular bodies (MVBs) (Thery et al., 2002). Proteins within the endocytic vesicles are seized and selectively allocated into the ILVs of MVBs. These MVBs can fuse with the plasma membrane to release ILVs, now called exosomes, into the extracellular space. The ILVs within MVBs can either be degraded by lysosomes or fuse with the plasma membrane to be released into the extracellular environment. These stable vesicles, can thrive in the blood, CSF, and urine.

Circulating exosomes are currently being studied as potential vehicles in intercellular communication. These nanovesicles contain molecules that reflect the molecular contents of the cell from which the exosome originated (Stern et al., 2016). These vesicles carry proteins, including heat shock proteins, RBPs, RNA, adhesion molecules, metabolic enzymes, and cytoskeletal proteins (Thery et al., 2002; Schorey and Bhatnagar, 2008). Exosome-associated proteins also play roles in determining exosome fate. Currently, exosomes are considered as a potential vehicle for the transmission of the toxic forms of tau (Jia et al., 2019; Sun et al., 2019; Nam et al., 2020).

## Tau oligomers and exosomes

Extracellular migration of tau is crucial in the development and spread of tau pathology. Many studies have shown that exosomes can aid in the intercellular neuron-to-neuron transfer of tau seeds (Dujardin et al., 2014a; Polanco et al., 2016, 2021; Wang et al., 2017). Both normal and pathogenic states of tau have been shown to be associated with exosomes (Saman et al., 2012; Fiandaca et al., 2015; Polanco et al., 2021). This further indicates that exosomes may play a role in generating and spreading of misfolded proteins (Wang and Han, 2018). Studies have shown a close association of released tau with exosomes (Chiarini et al., 2017). Exosome-associated tau is detectable in CSF before the onset of neurodegenerative phenotypes associated with AD (Sengupta et al., 2017). In chronic traumatic encephalopathy (CTE), total plasma exosomes levels cases did not differ between experimental and control groups (Stern et al., 2016). However, CTE-positive groups had higher levels of tau-associated plasma exosomes. Additionally, tau has been shown to be secreted in association with ectosomes, which are within the same class of vesicles as exosomes. A recent study has shown that overexpression of 4R0N tau in neuroblastoma cells recruits proteins associated with the exosomal proteome (Saman et al., 2014). Due to the intracellular origin of exosomes, they may serve as a pathway for cytosolic neurodegenerative proteins to be released and spread.

Exosomes may serve as mediators for the delivery of toxic oligomer forms of tau to neighboring cells. After interacting with the plasma membrane surface, the transfer of cytosolic neurodegenerative tau into the recipient cells can occur. Once inside the neighboring cell, the misfolded tau protein may interact in a prion-like way with functional conformations of tau to induce misfolding. This fusion mechanism has been directly shown by the use of luciferin to mark for exosomes. The labeled exosomes directly interacted with the plasma membrane and delivered their intraluminal contents into the cytosol of targeted cells (Montecalvo et al., 2012). These findings suggest that exosomes can transport or mediate oligomerization in exosomes and deliver their toxic cargo to a recipient for the further propagation of tau pathology (Figure 2).

## Conclusion

The misfolding and polymerization of tau into oligomers is a major event in the pathogenesis of tauopathies. A large body of evidence suggest that these toxic intermediates act as seeds, inducing the misfolding and propagation to neighboring cells in a prion-like fashion. Likewise to prions, the transferred tau oligomers may induce the conversion of poorly structured monomeric tau into a β-sheet rich structures. Studies conducted *in vivo* have demonstrated that seeds of tau oligomers propagate endogenous tau oligomers formation and neurodegeneration. A growing body of evidence suggests the cell-to-cell transmission of toxic tau aggregates seems to occur *via* exosomes. These nanovesicles can diffuse across the blood brain barrier carrying toxic cargo, thus are potential therapeutic targets and biomarkers in tau pathologies. Understanding the dynamic of tau oligomers formation, release and uptake by neighboring cells is critical for the future development of treatments for tauopathies.

## Author contributions

NJ wrote the first draft. All authors contributed to manuscript preparation and revision and read and approved the submitted version.
